# On Diseases of the Liver

**Published:** 1845-10

**Authors:** 


### On Diseases of the Liver. By *George Budd,* M.D. F.R.S. Professor of Medicine in King's College, London, &c. Octavo, pp. 401. Churchill: London, 1845.

Dr. Budd states, as a reason for his having undertaken the present treatise, that "while the press has been teeming with works on the diseases of the Nervous System, of the Chest, of the Kidney, of the Skin, comparatively few have appeared, of late years, on diseases of the Liver. This, assuredly," he adds, "is not owing to any falling off in our sense of their importance, but to the vague and unsatisfactory state of our knowledge respecting them." We are glad that the task of supplying the deficiency has been taken up by so active and enlightened an enquirer as Dr. Budd, who has evidently turned to the best possible account all the advantages of his former situation of Physician to the Seamen's Hospital on board the Dreadnought, and of his present one of Physician to the King's College Hospital.

The work commences with an introductory exposition of the minute anatomy of the Liver, and of the uses of its important secretion—the Bile. The whole chapter will well repay a diligent perusal; for, although there is perhaps nothing new in the details that are given, the description throughout is clear without being wearisome. The following passage (although taken from a subsequent Chapter) presents a succinct and lucid view of the minute structure of the Hepatic parenchyma.

"We have seen that the lobules of the liver are spaces mapped out by the ultimate twigs of the portal vein, which are hairy, as it were, with capillaries springing immediately from them on every side, and forming a close and continuous network; and that the interstices of these capillaries are filled with nucleated cells. It is in these cells that the vital chemistry of secretion goes on. It is seen by the microscope that in different livers, the cells vary in size; that in some they are almost transparent, in others opaque, and apparently more solid; that in some they contain but a few very small oil-globules, while in others, they are distended almost to bursting with globules of oil; that in some, they are colourless or nearly so, and in others, yellow with bile; that in some specimens, again, they are broken-down and destroyed. It is probable, too, that in some cases the cells are only slowly reproduced; that, without complete destruction, they become less productive of new cells, so that at length the number of active cells is much diminished.

"These differences in the condition of the cells cause, of course, corresponding differences in the size, colour, and texture of the liver; differences, which were noticed long before that knowledge of the intimate structure of the organ was obtained, by which we are now enabled to explain them." P. 197.

The Biliary Secretion serves various most important purposes in the animal economy. One of these purposes is unquestionably that of purifying the blood from certain noxious and effete principles, derived from the continual waste of the body and the metamorphosis of its tissues. The Bile, it is well known, contains a very large proportion of Carbon; and it has therefore been very reasonably inferred that the Liver is one of the emunctories employed by nature for eliminating the superabundance of this element from the system. Viewed in this light, it may be regarded as a co-operating—and, under certain circumstances, a partially-compensating—organ with the Lungs and Skin in the process of decarbonising the blood.

But there is another way in which the Liver tends to maintain the purity of the vital fluid; and to this point we particularly invite the reader's attention, as the explanation bears upon the history of several of the pathological alterations to which the organ is subject, and which we shall afterwards have occasion to bring under his notice.

"It will be remembered that all the blood sent to the stomach and intestines has to pass through this organ, before it can again mix with the venous blood from other parts of the body. Now the blood that has come from the stomach and intestines must necessarily be charged with many impurities, besides those derived from the mere decay of the tissues. Along the extensive mucous tract with which everything we eat or drink is brought in contact, absorption is constantly going on, and various matters must, therefore, enter the portal vessels, not fit by their nature to form blood, or to serve any other purpose in the body. Many of these substances are removed from the blood in its passage through the liver. The discharge of such matters through the liver, when they are in unusual quantity, or of a particular kind, is, no doubt, the primary condition of many biliary disorders." P. 28.

Having discussed the several points connected with the Anatomy and Physiology of the Liver, the author proceeds to describe its various Morbid Conditions. These he has arranged in five chapters; of which, the *first* treats of Congestion; the *second*, of Inflammation and its Consequences; the *third*, of "diseases which result from faulty nutrition of the liver, or faulty secretion;" the *fourth*, of "diseases which result from some growth foreign to the natural structure;" and the *fifth*, of Jaundice. There is an Appendix, which contains a good account of the "Liver-fluke" in Man, as well as in the Sheep and other graminivorous Animals.

### *Congestion of the Liver.*

The description given by our author of this morbid condition is lucid and instructive: as a matter of course, he has drawn largely, for his illustrations of the changes produced by its presence, from the admirable paper of Kiernan in the Philosophical Transactions. The most frequent cause of Hepatic Congestion is the existence of some obstruction to the free return of the venous blood to the right side of the Heart, in consequence of cardiac or pulmonary disease.

"It often happens," says Dr. Budd, "that in such persons, when the circulation is more than commonly impeded, the liver grows larger. Its edge can be felt two or three inches below the false ribs. If the circulation be relieved by bleeding, or by diuretics, or by rest, the liver returns to its former volume. This enlargement of the liver from congestion, often takes place, and again subsides, very rapidly, according to the varying conditions of the general circulation.

"In estimating the bulk of the liver, in congestion and other diseases, we must bear in mind, that its natural limits vary with posture and many other circumstances. It descends an inch or two lower when the person under examination is standing or sitting, than when he is lying down; it is lower after inspiration, than after expiration; and it may be pushed down by fluid in the cavity of the pleura, or by bloated, emphysematous lung." P. 41.

The complexion of patients labouring under Congestion of the liver, induced by the cause now mentioned, is usually purplish with an admixture of dingy yellow: the blood is not only imperfectly decarbonised, but is also not completely freed from the principles of the biliary secretion. When the congestion continues for a considerable length of time, the proper secretory apparatus (the nucleated cells) of the liver seems to be more and more injured, until perhaps it is irretrievably damaged; and then the secretion of the bile ceases almost entirely to be effected. Several cases are related by Dr. Budd, "where, from the flow of bile having been long obstructed by the closure of the *ductus choledochus*, the liver had entirely lost its lobular appearance and contained no nucleated cells; so that, when a portion of it was examined under the microscope, nothing was seen but free oil-globules and irregular particles of greenish or yellow biliary matter." The same effect may probably arise from any serious lesion of the extreme vessels or biliary tubules; as, for example, in the hardened and granular state of the liver so frequent in drunkards, to which the appellation of *cirrhosis* has been applied.

Besides the mechanical and chronic causes of Congestion of the liver, to which we have alluded, this viscus is liable to become engorged from some of a more active and vital nature. In *ague* for example, the liver, like the spleen, is apt to be very much enlarged from sanguineous accumulation, during the continuance of the febrile paroxysm. In *purpura* also, and indeed in most diseases in which the blood is in a dissolved state, hepatic congestion is liable to occur. We need scarcely say that the nature of such congestions, as well as the proper method of relieving them, must be very different from that morbid condition which is induced by a stagnation of the blood in the parenchyma of the liver from organic disease of the heart and lungs.

From Congestion we pass on to Inflammation of the Liver. The character and features of this morbid condition vary very much, according to the exciting cause of the disease, the parts of the hepatic structure that are chiefly affected, and the nature of the patient's constitution. Dr. Budd arranges the inflammatory diseases of the liver under five heads:—1. *Suppurative* inflammation; 2. *Gangrenous* inflammation; 3. *Adhesive* inflammation; 4. Inflammation of the *veins;* and 5. Inflammation of the *gall-bladder* and *ducts*. It might be very easy to object to such a classification, which is founded, it will be observed, on two distinct principles—the *nature* of the morbid action, and its particular *locality* or seat. The only change, however, which we shall make, is to put the third species enumerated in the place of the first.

*Adhesive Inflammation of the Liver*:—or inflammation attended with the effusion of Coagulable Lymph. This effusion may be either on the surface, or in the parenchymatous substance, of the organ; but it is more particularly to the latter of these states that we wish to call the reader's attention. The following description of the effects of this lesion—so frequently induced by the use of spirituous liquors—deserves his notice.

"Deep-seated adhesive inflammation of the liver produces different effects, according to the parts it principally involves. Sometimes the lymph is effused almost exclusively into the areolar tissue in the portal canals of considerable size, and if the person die long after this has occurred, all the considerable branches of the portal vein are found surrounded, in some places to a distance perhaps of half an inch, by new fibrous tissue, which by its contraction has drawn in and puckered the adjacent portions of liver. The remaining portions of liver may be little, if at all, altered in texture, and may be readily scraped away from these indurated portions. The main branches of the vein are pervious, but many of the small twigs that spring from them are obliterated. The parts which these twigs supplied are atrophied, and the liver proportionally reduced in bulk. Where such portions are near the surface, the capsule is somewhat drawn in and puckered. Together with these changes, there are usually, if not always, thick false membranes on the capsule of the liver, or extensive adhesion, by means of old tissue, between the liver and adjacent organs. Usually, too, there are old false membranes on the surface of the spleen, and marks of adhesive inflammation of other parts, especially the pericardium and the pleura.

" I have several times met with this form of disease in persons who had drunk hard of spirits. It comes on with well-marked symptoms of inflammation of the liver,—pain in the side, vomiting, fever, and perhaps jaundice. These symptoms subside after a time, but the patient does not regain his former health. The liver has been permanently damaged; part of its secreting substance becomes atrophied from closure of the small portal veins, and it is no longer adequate to its office. The patient has difficult digestion, looks sallow, and does not recover his former strength." P. 108.

The deposition of Lymph or fibrous tissue around the smaller branches of the vena portæ, that separate the lobules of the liver from each other, renders its parenchymatous substance much tougher and more indurated than in health. When we make a slice of it, we observe thin fibrous lines interposed between small irregular masses of the lobules. At those parts on the surface of the viscus, which correspond to these lines, its investing capsular membrane is puckered or drawn in; producing that " hob-nailed" appearance that has been of late so much spoken of.

" The tissue of the liver is paler than natural, from the presence of this white fibrous tissue, and from its containing but a small quantity of blood; and it is often yellowish from accumulation of biliary matter in the cells. When this is the case, a section has the greyish and yellow colour of impure bees-wax, and, in consequence, the disease has been called by the French, *cirrhosis*—from κιῤῥος, yellowish.

" In other cases again, the quantity of this adventitious fibrous tissue is much greater, and by its contraction the lobular substance of the liver is drawn into round nodules, which being of a deep yellow colour from accumulation of biliary matter, are in strong contrast with the gray fibrous tissue between them.

" This state has been described by Abercrombie, who says, the yellow matter of cirrhosis is sometimes in small nodules, like peas, dispersed through the substance of the liver. He adds, ' A case is described by Clossy, in which the structure of the liver was wholly constituted of a congeries of little firm globules, like the vitellarium of a laying hen; it occurred in a boy of fifteen, who had immense ascites. In a case by Boisment, these nodules were as large as peas, and the liver was much diminished in size; the case was chronic with ascites. The French writers have a controversy whether the cirrhosis or yellow degeneration of the liver be a new formation, or a hypertrophia of the yellow substance, which they suppose to constitute a part of the structure of the liver in its healthy state. No good can arise from such discussions, as it is impossible to decide them.' (Diseases of the Stomach, &c., 2nd edition, p. 369.)" P. 109.

In the early stage of Cirrhosis, (the *gin-drinkers' liver* of English writers) the size of the viscus is usually enlarged, in consequence of the deposition of serum and lymph throughout its substance. The thinner and more watery part of the effusion is, however, gradually absorbed in time, the fibrine contracts, the small twigs of the portal veins, and their accompanying biliary tubules, are compressed by the new tissue, and the lobular substance of the liver, receiving less blood than it should do, wastes. On all these accounts the liver diminishes in size; and in protracted cases, from the small quantity of blood it contains, and the great atrophy of the lobular substance, it is usually very much smaller than in health.

As, in Hepatitis arising from the excessive use of ardent spirits, the physical and material cause of the inflammation is conveyed directly by the portal vein to the parenchyma of the liver, we are at once prepared to expect that the morbid change should be most conspicuous in it: the capsule or investing membrane is only secondarily affected.* Even in some cases of "hob-nail" liver, the peritoneal coat remains sound, and the *tunica propria* is so little, if at all, altered, that it may be readily detached and stripped off. But this exemption from morbid change is certainly not very common.

Associated with Cirrhosis of the Liver, we not unfrequently find more or less serious organic Disease of the Heart. M. Becquerel—in an elaborate paper upon the subject, published in the Archives Generales for 1840—states that the heart was diseased in twenty-one out of forty-two cases of Cirrhosis, of which he has given an analysis: he is of opinion that in these cases the heart was diseased before the liver. We think that Dr. Budd is quite justified in withholding his assent to this position, and in maintaining that "obstructed circulation through the chest has no *direct* influence in causing the disease (cirrhosis), and that it contributes to it only by giving greater effect to the influence of alcohol and other efficient causes."

The pernicious influence of spirit-drinking in inducing liver-disease is much promoted by exposure to the vicissitudes of the weather—the prolific cause of so many insidious inflammatory affections among the lower classes—by a residence in a hot climate, and by whatever is calculated to disturb the healthy function of the chylopoietic organs, and to vitiate the products of digestion. In a good many of the published cases of Cirrhosis, organic affection of the stomach was present.

It is scarcely necessary here to allude to the *symptoms* of Adhesive Inflammation of the Liver, and of its consequences; more especially as they vary so much according to the acuteness of the attack, and the stage or period of its existence. The presence of pain and tenderness over the region of the liver, in a spirit drinker, should always be viewed with suspicion, and carefully watched. When the organ has become seriously diseased, the complexion is always more or less jaundiced, and the skin is dry and rough. There is moreover in the majority of cases a tendency to Ascites, and not unfrequently also to various Hæmorrhagic phenomena,

---

* Dr. Percy of Birmingham has stated in his prize Essay, published a few years ago, that, in dogs poisoned by alcohol, he could recover the spirit from the blood, the brain, and various other organs, but in greatest quantity from the liver.

as bleeding from the nose or rectum (piles are not unfrequent), the appearance of purpuric spots on the surface, and so forth.* There is a circumstance connected with the dropsical effusion arising from this cause that deserves our notice, viz: that the Ascites very often exists alone and without œdema of the legs or of any other part; and that, when there is such œdema present, it almost always takes place subsequent to the Ascites. The cause is obvious; it is the Portal circulation which is at first chiefly, and perhaps only, affected.

*Treatment.*—In the early stage of the disease, when there is pain and tenderness in the hepatic region, *cupping* over this part, and the use of saline medicines, with low diet, are in general the best remedies. General bloodletting is seldom admissible in hard drinkers: delirium tremens is apt to occur after the loss of much blood. When bleeding is not required, or is not deemed safe, the application of a *blister* will generally afford decided relief. Dr. Budd says that, when the fever has abated, and the liver is still very large, *mercury*, and the *iodide of potassium*, are the medicines from which most benefit may be expected. We would not wait for the abatement of the fever, before we gave the former, at least, of these most potent remedies. A couple of grains of *calomel*, or three or four of—that excellent preparation—the *hydrargyrum cum cretà*, either alone or in combination with the *extract of colchicum*, may be administered night and morning, from the very first setting in of the hepatic uneasiness. Subsequently the *Plummer's pill* is a most valuable alterative. When ascites has supervened, we must avoid all lowering treatment. Hydragogue cathartics exhaust the strength, without materially diminishing the dropsical effusion; and surely long courses of mercury can do little or no good, when the parenchymatous structure of the Liver is deeply and permanently altered. An occasional warm or tepid bath is always grateful to the patient's feelings, and will often serve to promote the urinary, as well as the cutaneous, excretion. Mild diuretics, with small doses of the *iodide of potassium*, are sometimes very useful; at least temporarily so.

### *Suppurative Inflammation of the Liver.*

The causes of this serious lesion are various.

1. It may follow a blow or other mechanical injury on the side. Andral has related a case of this sort: but the occurrence is certainly very rare.

2. A far more frequent cause of Abscess of the Liver is suppurative inflammation of some vein, and the consequent contamination of the blood with pus. It is well known that what have been (not very correctly) called "metastatic abscesses" are apt to occur after injuries, surgical operations, &c., in various internal organs. Of these the Liver is one, in which they very frequently occur—more frequently indeed in it than in any other,

* Another symptom is an enlarged or varicose state of the veins on the surface of the abdomen—arising from the impediment to the free return of the blood along the deep-seated vessels. This symptom is often observed in different organic diseases of the abdominal viscera.

the Lungs alone excepted. Dr. Budd discusses with much clearness the general question as to the formation of these abscesses; and he comes to the conclusion, that their immediate cause is the presence of some irritating substance conveyed to the affected parts by the blood, and that in most instances the irritating substance is Pus, derived from the inflammation of the inner surface of a vein. "The globules of the purulent matter, mingled with the blood, are conveyed to the capillary vessels of the lungs, and, it would seem, by becoming mechanically arrested there, excite each circumscribed inflammation and abscess. If any of the globules pass through the capillaries of the lungs to the left side of the heart, they are sent in the arterial current to other organs, and becoming arrested in the capillaries of these organs, excite, as in the lungs, inflammation of limited extent, rapidly passing on to abscess."

It has been long well known to surgeons that Injuries of the Head are often followed by abscesses of the Liver. No rational explanation of the fact could be given, until of late years.

"From the researches of Mr. Arnott in this country, and of MM. Dance and Cruveilhier in France, no doubt remains that the abscesses in such cases result from suppurative inflammation of a vein, either in the soft parts, or between the tables of the skull.

"Many false theories of the mode of formation of the abscesses of the liver, consequent on injuries of the head, have been maintained under the erroneous impression that abscesses exist in the liver only. It was, however, long ago remarked by Morgagni, that, in these cases, there are often abscesses in the lungs, heart, spleen, and other organs, as well as in the liver. The abscesses in the liver attracted more attention than those in the lungs, on account, perhaps, of their larger size, and their being more conspicuous from the stronger contrast between the colour of pus and the natural colour of the organ." P. 53.

In the cases to which we have hitherto alluded, the *peccant* cause must have passed through the capillaries of the Lungs, before it had reached any other organ; and hence it is that these metastatic abscesses are so much more frequent in the lungs than in the other viscera of the body. But, if the seat of the suppurative Phlebitis happen to be one of the veins that go to form the Vena Portæ, the pus, it is obvious, will be carried first to the Liver, before it be conveyed elsewhere; and then the abscesses will be found alone, or in greatest number, in that organ. The results of several experiments on animals have served to illustrate this important point in pathology. For example, Cruveilhier found that, if mercury be injected into one of the mesenteric veins, it will be all stopped in its course through the Liver, and will cause circumscribed abscesses throughout its substance, just in the same manner as it does in the Lungs when it has been injected into the crural vein. Now the same result is apt to follow in the human subject, when any of the mesenteric veins become inflamed; as they will sometimes do after operations upon the Rectum, or for strangulated Hernia. Dance has related several such cases; and in these, as well as in the instances related by M. Cruveilhier, it is especially worthy of notice, that no abscesses were found in any other viscus or organ; all the pus, furnished by the inflamed veins, thus appearing to be stopped in its passage through the Liver.

Keeping these things in view, we are not surprised to find that Dr. Budd is inclined to believe that "by far the most frequent cause" of Abscess of the Liver is ulceration of some part of the alimentary canal, or of the gall-bladder or ducts—the veins of which parts all go to the portal system. Of 29 cases recorded by Annesley, there was Dysentery and ulceration of the large intestine in no fewer than 21; and, in two others, the gut exhibited traces of a former lesion. Again, out of thirteen cases observed by Dr. Budd in the Seaman's Hospital, on board the Dreadnought, ulcers were found in the large intestine or stomach, in nine.* That there is an intimate connexion between the intestinal lesion in Dysentery and the formation of Abscesses in the liver cannot be fairly questioned, although the accurate Abercrombie seems to regard the co-existence of these diseases as accidental.—(*Dis. of the Stomach, &c.*, 2nd ed. p. 266.) Annesley, while admitting that Hepatic Abscess is in a good many cases connected with previous Dysentery, very candidly confesses that he is unable to point out any definite or established relation between the two maladies. In some cases, the Dysentery seems to be the direct consequence of the disease of the Liver; while, in others, the Hepatic and Intestinal diseases seem to be so nearly coeval, that it is impossible to say which existed first. Dr. Budd seems inclined to push his generalisations a little further; for we find him not hesitating to declare his opinion, that "in all the cases, or most of the cases, in which abscess of the liver and dysentery are associated, the former disease is the consequence of the latter." The data, which we as yet possess, scarcely warrant this opinion in its full extent. The *modus operandi* of the one disease in inducing the other is thus simply and clearly explained by our author.

"Admitting dysentery, or ulceration of the bowel, to be a source of abscess of the liver, it is obvious that the liver does not become involved by spreading of the inflammation, but by some contamination of the portal blood.

"This may be either by *pus*, formed by suppurative inflammation of one of the small intestinal veins; or by matter of other kind resulting from softening of the tissues; or by the fetid gaseous and liquid contents of the large intestine in dysentery, which must be absorbed and conveyed immediately to the liver. It seems probable, that contamination of the first kind usually gives rise to small scattered abscesses; of the last, to diffuse inflammation, and a larger, perhaps single, collection of pus. If the morbid matter be such that it does not mix readily with the blood—as globules of pus or mercury—it will cause small, circumscribed abscesses, the rest of the liver being healthy. If, on the contrary, the morbid matter be readily diffusible in the blood, all the blood will be vitiated, and diffuse inflammation result." P. 64.

Ulceration of the Stomach is not unfrequently accompanied with the formation of Abscess in the Liver. The subject of the connexion between these two morbid states has not hitherto met with that attention from

---

* We may refer to Welb's *Pathologia Indica*—of which there is an ample review in the last number of this Journal—as affording additional testimony on this point. The author gives a tabular view—drawn up by Mr. Geddes of Madras—of 28 cases of Hepatic Abscess; in 19 of these, either Dysentery, or something very much like it, seems to have been present.

pathologists which it deserves:* but the cases, adduced by Dr. Budd, are quite sufficient to convince every one of the important fact, that organic disease of the stomach is liable to be attended with suppurative disease of the hepatic parenchyma. Nearly the same thing may be said of Ulceration of the Gall-bladder or ducts. On this point we find the following instructive remarks:—

"The ducts, the gall-bladder, and the capsule of the liver, are nourished by the hepatic artery, and blood flows, not from the portal vein to them, but from them to the portal vein. This circumstance explains how ulceration of the gall-bladder, like ulceration of the stomach or intestines, may cause abscess of the liver; and it also explains the fact, noticed by many physicians who have written on abscess of the liver, that in this disease the gall-bladder, the large ducts, and the capsule, are seldom involved. The suppurative inflammation is confined to those parts of the liver that receive blood from the portal vein. The frequent absence of every trace of inflammation of the capsule in cases of abscess of the liver has been expressly noticed by Annesley and by Dr. Stokes, as very important in reference to treatment." P. 69.

The facts, which we have quoted, will suffice, we think, to prove the important pathological position, that the formation of Hepatic abscesses is very frequently associated with the existence of ulceration in some one part of the alimentary canal; but it must not be forgotten, that there are certain *kinds* of this intestinal lesion which are very rarely, if ever, attended with any decided change in the substance of the liver. Thus, it is very rare in cases of Typhoid Fever, or of Phthisis—in both of which diseases, ulceration of the bowels is so commonly met with; nor was it found to be present in any of the ten cases of ulceration of the duodenum after Burns, related by Mr. Curling in his paper in the Medico-Chirurgical Transactions for 1842: (vide the No. of this Journal for January, 1843). The converse, too, of the proposition is equally true; viz. that Abscess of the Liver may exist—more especially in tropical climates—without any co-existing ulceration of the bowels. Our author, indeed, seems rather unwilling to admit this fact, and he is every now and then suggesting that there may have been a cotemporaneous lesion of the intestines even in those cases where it has not been observed. He is no doubt quite correct in asserting that the Hepatic congestion, which arises from an impediment to the free return of the venous blood in consequence of some pulmonic or cardiac disease, seldom, if ever, produces suppurative inflammation; nor is this morbid state at all a common result of spirit-drinking, as has been alleged by some writers; but the accuracy of the opinion, put forth in the following extract, may, we think, be very fairly questioned. After stating that, in India, great influence is attributed to the mere heat of the climate in causing inflammation and abscess of the Liver, he goes on to say:—

---

* It is but justice to Broussais to remark, that it was he who first pointed out, or at least insisted upon, the intimate dependence of Hepatic disease upon gastro-enteric irritation. Dr. Stokes has treated this important subject with his accustomed ability in his valuable essays upon Gastro-Enteritis and Hepatitis in the Cyclopædia of Practical Medicine.

" Another cause, brought forward to explain the frequency of abscess of the liver in India, is remittent or intermittent fever, or, more correctly, the malaria that produces these fevers. It seems established, that in some of these fevers, the liver, like the spleen, becomes congested, and much enlarged in consequence; and in yellow fever and the severe forms of remittent fever, it is much and permanently damaged in its secreting element. Yet it may be doubted whether suppurative inflammation of the liver takes place in these cases without ulceration of the stomach, or gall-bladder, or intestines, which so often occurs in some climates in the course of the severe forms of marsh fever." P. 73.

In a subsequent passage, however, he remarks :—

" It may be, however, that in some parts of India, a peculiar malaria, favoured perhaps by the heat of the climate, produces abscess of the liver independently of ulceration of any part of the mucous surface that returns its blood to the portal vein. We know that marsh-fevers differ very much in type, and damage different organs in different seasons and climates; and even according to different degrees of concentration, merely, of the poison by which they are produced." P. 73.

Abscesses of the Liver sometimes attain an immense size. In a case observed by Dr. Budd, the quantity of purulent matter was estimated at two quarts; in one related by Annesley, there were not less than 90 ozs.; and Dr. Inman, of Liverpool, has sent to our author the account of one still more extraordinary, in which the quantity of matter was found by measurement to be 13 pints. The abscess, it is well known, may burst either outwardly, or inwardly into the lung or pleura, or into an intestine, or into the cavity of the abdomen.*

With respect to the *symptoms* of Suppurative inflammation of the Liver, it is quite unnecessary to make many remarks. They are very obscure in not a few cases, and the lesion is often discovered only after death, without perhaps having been ever suspected during the life of the patient. There may be no pain in the side or shoulder, no vomiting, nor yet any appearance of jaundiced discolouration. Andral, Abercrombie, &c. have noticed this, not unfrequent, absence of any characteristic symptoms; and Annesley, one of the highest authorities that can be quoted, expressly says: "The formation of matter (in the liver) may commence and terminate without the appearance of any of those signs on which the inexperienced are taught to rely." This experienced writer has made a remark respecting the *pain in the right shoulder*, (which is usually regarded as characteristic of liver-disease,) that deserves notice. According to his experience, the presence of this symptom uniformly indicates that the disease is seated in the *right* lobe of the liver. There is good reason to believe also, that the pain is always much more uniform and decided, when the

---

* " It has been supposed," says Dr. Budd, " by some medical men in India, that the pus in an abscess of the liver may be absorbed, and eliminated, *as pus*, in the urine. But this notion is evidently erroneous. Pus-globules, from their large size, cannot directly enter the blood-vessels or escape from them. The matter in the urine, supposed to be pus, was probably a deposit of phosphates. During the severe constitutional disorder that attends purulent phlebitis, there is often a sediment of this kind in the urine,—having to the naked eye much the appearance of pus, but under the microscope, showing, instead of pus-globules, beautiful phosphatic crystals."

convex surface, that when other parts, of the viscus are affected. MM. Louis and Andral have not hesitated to declare it as their opinion, that pain in the right shoulder is not a frequent symptom of hepatic disease: although far from being constant, it is nevertheless present in a sufficiently large proportion of cases, to entitle it to a place among the diagnostic signs. The former of these gentlemen has gone so far as to express his belief that the *vomiting* and *cough*, that are frequent attendants upon diseases of the Liver, are dependent, the first upon a *gastrite*, and the latter upon a *bronchite:* such statements are at utter variance with the experience of the most practical and trustworthy writers upon the subject. The tension or *rigidity of the right rectus muscle*—a symptom first insisted upon by the late Mr. Twining—is found to exist in other diseases of the liver, besides suppurative inflammation: it is one of those signs that should always be sought for by the physician. Symptoms may sometimes be derived from the operation or effects of medicines; and in this way the diagnosis of a disease may, in some degree at least, be determined. If, for example, a pain yields to steel or bark, we suspect that it was truly neuralgic; if to alkalis and colchicum, we presume that it was of a gouty nature; and if to bloodletting and purgatives, we have fair reason to believe that it was congestive or inflammatory. Now there is something, in reference to the action of Mercury in Hepatic inflammation, that well deserves the notice of the practical physician. It has been found by all the most experienced observers that the preparations of this mineral almost invariably fail of producing its accustomed constitutional effects, when suppuration has once taken place. Annesley says: "There can be no doubt that the system will not be brought under the full operation of Mercury, or that ptyalism will not follow upon the most energetic employment of this substance, where abscess exists." He repeats this opinion again and again, and even considers resistance to the action of Mercury as a proof that abscess has formed in the liver. It may be laid down as an invariable rule, that this mineral should never be administered (except indeed for the relief of an occasional symptom) when there is reason to suspect that suppuration has taken place: it can then do no possible good, and will only serve to exhaust the patient's strength, and exasperate, by its depressing influence, the local malady.

Passing over Section IV., which treats of *Adhesive and Suppurative Inflammation of the Portal and Hepatic Veins*, we come to the following one, which it will be observed, from the heading affixed to it, contains a variety of matter:

"*Inflammation of the gall-bladder and ducts—Catarrhal and suppurative inflammation—Croupal, or plastic, inflammation—Ulcerative inflammation—Effects of ulceration of the gall-bladder and ducts—Effects of permanent closure of the cystic and common ducts—Fatty degeneration of the coats of the gall-bladder.*"

Marks of Inflammation and other disease are much more common in the gall-bladder, and in the cystic and common biliary ducts, than in the hepatic ducts. The following case from Andral's Clinique (t. iv. p. 499) is regarded by our author as an example of acute inflammation of the common duct.

"In the summer of 1824, a man, about 30 years of age, felt severe pain in the right hypochondrium for two days, and then became jaundiced. When he entered the hospital, the jaundice and the pain were still present; and immediately below the cartilages of the false ribs, was a moveable, pear-shaped tumour, which Andral took for a distended gall-bladder. The pulse was quick, the skin hot, the bowels obstinately bound. (Twenty leeches to the anus; enemata; foot-baths; barley-water.) The next day, the fever ceased. During the three following days, the tumour grew less, and then disappeared together with the pain. The jaundice went off, the constipation ceased, and the patient soon left the hospital well." P. 158.

The symptoms of such an affection may reasonably be supposed to be pain in the situation of the duct, followed, at the end of two or three days, by jaundice and distention of the gall-bladder; fever; constipation, and probably nausea and vomiting. The case may readily be mistaken for one of inflammatory jaundice; but our diagnosis will be materially assisted by the circumstance of the pain being limited to a small spot in the situation of the common duct, and by *the early appearance of a large, moveable pear-shaped tumour*, produced by the projection of the distended gall-bladder. If this tumour be very painful and tender, we may suspect that the gall-bladder itself is inflamed. Dr. Graves, in his recent work on Clinical Medicine, has related a well-marked example of plastic or croupal inflammation of this organ. In a young woman, who died with the symptoms of decided Jaundice (there was no pain in any part except *at a point between the right hypochondrium and epigastrium, greatly increased by pressure*), the following appearances were found on dissection. The substance of the liver itself exhibited nothing decidedly abnormal; but "the gall-bladder was distended, and, on being opened, was found completely filled by a dark green mass of a tenaceous viscid nature, apparently lymph. This substance was of the same pyriform shape as the gall-bladder, and terminated by its narrow extremity at the commencement of the gall-duct. On its removal, the lining membrane of the gall-bladder presented a bright scarlet colour and villous appearance, and the natural and beautiful 'honey-comb' arrangement of the mucous membrane was completely effaced. There was no softening or ulceration of the membrane, nor was the colour different in any part. It resembled very much the appearance of the mucous membrane in acute laryngitis. The walls of the gall-bladder were much thickened. There was no obstruction in the ductus choledochus, the cystic or hepatic ducts, and their lining membrane was quite free from any unusual vascularity: the duodenum and stomach were stained with the colouring matter of the bile, but in other respects were healthy; no gall-stones or other obstruction; the kidneys were natural."

A frequent cause of Inflammation of the Gall-bladder, and of the cystic and common ducts, is the mechanical irritation of gall-stones. On the other hand, the thickening of their parietes and the consequent contraction of these ducts must necessarily impede the free exit of the bile, and thus tend to favour the formation of these productions.

*Ulceration of the Gall-bladder* is not unfrequently met with in patients who have died of remittent fever. Sir G. Blane has noticed this appearance in his account of the Walcheren fever; and Mr. Boyle, in his description of the Sierra Leone fever, says "there were, in almost all cases, traces of inflammation in the pyloric extremity of the stomach, extending thence along the duodenum to the entrance of the gall-duct, about which, for the space of a Spanish dollar, the inflammation seemed to have attained the greatest height. The duct was ordinarily choked by dark-coloured, viscid bile."

In this country, ulceration of the gall-bladder is perhaps most frequently connected with the presence of gall-stones; but a more common result of the presence of these substances is *closure of the cystic*, or even of the *common biliary duct*. The former of these lesions may exist without any necessary symptoms of hepatic disorder; the latter, as a matter of course, invariably induces incurable jaundice, and eventually certain death. When the obstruction of the common duct takes place gradually, the gall-bladder and duct sometimes acquire an enormous size. Abercrombie cites from Boismont a case, in which the hepatic gall-ducts became so distended, and the lobular substance of the liver was so wasted at the same time, that this viscus had the appearance of a large undulating cyst.

The change in the parenchymatous substance, under such circumstances, is very remarkable. The proper nucleated cells of the liver, which serve to secrete the bile, are damaged or totally destroyed, the capillary vessels waste, and the whole organ in consequence shrinks and no longer presents any appearance of distinct lobules. Dr. Thomas Williams has given an interesting account of these changes in his most valuable paper " On the Pathology of Cells" in Guy's Hospital Reports. (The reader will find an analysis of this paper in the Number of the Medico-Chirurgical Review for January, 1844). According to this gentleman's observations, the nucleated hepatic cells entirely disappear; and nothing more than minute free fatty particles, and equally free, floating, amorphous granular matter are anywhere discoverable. In a case carefully examined by Dr. Budd, the appearances of the lobular substance entirely coincided with those so faithfully described by Dr. Williams; the liver being entirely " made up of vessels and the areolar tissue connecting them, with free oil-globules and solid particles of yellow and orange biliary matter that were left when the watery and more soluble parts of the retained bile had been absorbed." In these cases of closure of the common duct, there is sometimes present (what we should at first not expect) a voracious appetite—arising most probably, as in Diabetes, from the imperfect digestion of the food. Hæmatemesis is a not unfrequent symptom of this, as well as some other, forms of chronic Hepatic disease: the cause is obvious; the circulation through the portal vein is more or less impeded, and there is consequently a back pressure upon the current of blood in the gastric and mesenteric veins.

In some, but certainly not in all, cases of obstructed excretion of the bile, the Brain suffers; and delirium, or coma and convulsions, precede the fatal issue. But such cases do not invariably prove fatal, at least for a very considerable period of time. Patients have been known to survive for several years in a jaundiced state, when there was reason to believe that the *ductus choledochus* had become closed, and the parenchyma of the liver was in a great measure atrophied at the same time. It is from such instances as these, where not only the *excretion*, but the *secretion* also, of the bile must have been suspended for a lengthened period, that it has been reasonably inferred that the Hepatic functions are not invariably and absolutely necessary to life.

"The destruction of the secreting element of the liver proves fatal, however in the end, by impairing nutrition and causing gradual but progressive wasting." The prolongation of life, under such circumstances, will depend much on the healthiness of the other chylopoietic viscera, the nature of the food taken, the state of the respiratory and cutaneous functions, the condition of the bowels and so forth. The steady use of ox-gall, in the form of pills, after each meal, may serve very materially as a substitute for the natural secretion; and frequent tepid bathing of the surface will always conduce to the comfort of the patient.

There is a very dangerous form of *Jaundice, depending*, it would seem, *upon a sudden suppression of the biliary secretion*, and accompanied with symptoms of alarming cerebral disorder. Dr. Alison published two well-marked cases of this formidable disease in the Edin. Med. and Surgical Journal for 1835. The following heading of the first case gives a brief summary of its chief features.

"*Occasional complaint of pain and heat in the abdomen, with thirst and chilliness, for seven weeks; then jaundice, followed at the end of two days by delirium—No tenderness of abdomen, or fever—Occasional singultus—Stools bilious—Coma—Purpuric spots on the skin—Death ten days from the occurrence of jaundice—Liver of a light yellow, smaller than natural, flabby—Mucous membrane of the ducts unnaturally white.*"

Dr. Bright also has ralated two cases of the kind in Guy's Hospital Reports. The first is headed thus:

"*Abdominal pain—Jaundice—Tenderness at the epigastrium—Occasional sickness—Three weeks after the appearance of jaundice, indistinct utterance, and loss of power in the left hand—soon followed by coma and death—Liver very small, soft or flaccid, and of a reddish-yellow colour—No marks of inflammation on the capsule or in the ducts, which were not even stained with bile—Brain congested.*"

Rokitansky enumerates the following signs or features as characteristic of this affection. "It is distinguished, during life, by its acute course,—extreme pain of the liver (by no means a constant or even a very frequent symptom),—nervous symptoms and jaundice—and, finally, a fatal issue amid fever, symptoms of blood-poisoning, irritation of the brain and its membranes, hydrocephalic softening of the brain, exsudation and softening processes generally, and especially of the mucous membranes, pneumonia, &c. The blood in the larger vessels of the liver is thin, fluid, of a dirty red-brown colour."

Unquestionably many of the symptoms may be fairly attributed to a poisoning or contamination of the blood from the non-elimination of the principles that are discharged, in the healthy state, by the liver. Dr. Budd appears to be of opinion, that the proximate cause of this malady is a serious lesion or destruction of the proper hepatic nucleated cells; but this can be regarded as merely a conjecture. The attack, in some cases at least, has more of the characters of a fever, accompanied with a peculiar disturbance—a sort of acute paralysis, if we may use such an expression—of the biliary functions, and subsequently with symptoms of alarming head-affection, than of a disease dependent upon any organic lesion or change of the hepatic structure. Whatever view we may take of this pathological question, the important practical point cannot be too strongly urged upon the attention of all medical men, that the supervention of excessive drowsiness, or of any other symptom indicative of cerebral oppression or irritation, in a case of acute Jaundice, must always be viewed with great suspicion, and as requiring most prompt and energetic treatment. Active purgatives, accompanied in some cases with the detraction of blood, are the principal remedies.

Among the various causes that are liable to give rise to Jaundice, arising from a suppressed secretion of the bile, may be enumerated, deep grief, or any violent mental emotion, the bite of serpents and poisonous insects, the miasm of certain fevers, the noxious products of depraved digestion, and certain vegetable or mineral poisons. In some cases, it is scarcely possible to discover any probable cause. Abercrombie has related an interesting case, in which there was a sudden occurrence of deep jaundice, accompanied subsequently with frequent vomiting of black matter, and great prostration, so that the patient sunk after an illness of three weeks, but without any symptoms of head-affection having made their appearance. On dissection, the liver was much diminished in size, of an almost black colour, and soft and disorganised throughout, like a mass of coagulated blood. The gall-bladder was empty and collapsed. Dr. A. calls it a case of "*black ramollissement of the liver.*" This state is unquestionably not the result of any inflammatory action ; it is more allied to Gangrene.

That the liver sometimes, although rarely, becomes the seat of *gangrene* is now admitted by all pathologists. Rokitansky says that he has, on several occasions, observed Gangrene of the Liver, in *connexion with gangrene of the lungs;* but that he has never found it without gangrene of some other part. Our author relates a most interesting case—which accords with, and confirms, this remark of the Austrian pathologist—of what may be called "gangrenous infection." It occurred in the practice of Mr. Busk on board the Dreadnought, and is headed thus:

"*Mortification of the toes from cold—Removal of the dead parts—Severe rigors followed by typhoid symptoms—Death on the sixth day—Gangrene of the liver, the lung, and the spleen; Necrosis of the thyroid cartilage; Ulceration of the pharynx; pus in the shoulder-joint.*"

The comments, appended by our author to the report of the case, are so interesting that we have great pleasure in submitting them to the reader's attentive perusal.

"In this case, the existence of gangrene, both in the liver and in the lung, was clearly shown by the defined line surrounding the gangrenous portions.

"The source of the mischief here was, no doubt, the gangrene of the toes produced by cold. The man was in the prime of life, of spare habit, muscular, florid, and in good health at the time of the frost-bite. The case shows us what a serious thing a small patch of gangrene in any part of the body may become.

"The dissemination of the gangrenous masses—the existence of a number of them *isolated and at a distance from one another*—proves that the septic agency was conveyed by the blood. The noxious matter thus disseminated destroyed the vitality of the tissues on which it acted most strongly.

"The chemical theory of these septic changes is now well known. All parts in which they are taking place, have a tendency to affect other parts brought into contact with them, with the same mode of transformation. The case just related, —and it is by no means a solitary one,—offers one of the most interesting illustrations of this theory in the whole range of pathology. But, whatever be the explanation adopted, the fact is certain, and it is one of extreme importance, that gangrene of the extremities, or of any part of the surface of the body, produced by cold, by pressure, or in any other way, has a tendency to infect other and remote parts of the body with the same change.

"The occasional occurrence of gangrene in remote parts of the body in low fevers, after sloughing of the skin of some one part has been caused by pressure, was particularly noticed by Dr. Graves, in his remarks on an interesting case in which gangrene of the lung was consequent on sloughing of the sacrum from pressure." P. 102.

The second Section of the Chapter, upon which we are at present engaged, is devoted to the consideration of *Fatty degeneration of the liver—partial deposit of fat in its substance—waxy liver—and the appearances caused by deficiency of fat in the liver.*

It is to Mr. Bowman that we owe our most correct knowledge of the true nature of the first of these morbid states. According to this accurate observer, the greater portion of the superabundant fatty matter, present in the hepatic parenchyma, exists in the form of oil-globules within the proper nucleated cells of the affected organ. Even in the healthy state, these cells always contain some oil-globules; but, in this disease, the number and size of these is enormously increased. The accumulation of fatty matter is sometimes so great, that more than one-half of the bulk of the entire viscus—which, moreover, is generally a good deal larger than natural—may consist of it. The tissue of such a liver is pale, and generally of a light buff colour throughout, dotted with brown or red, the dots indicating the centres of the lobules; it is moreover soft, and greases the hands or the scalpel like common fat. When the quantity of oil is less, the liver presents the peculiar appearance so well known as the "*nutmeg liver.*" It may not feel greasy; but that it contains an unusual quantity of fat may be at once detected, by placing a thin slice of it on a piece of paper, and exposing it to the action of heat; some of the oil or fat exudes, and greases the paper. But the best way of ascertaining the morbid change is to examine a small particle of the liver with the microscope. The early stage of *Cirrhosis,* it may be remarked, is apt to be mistaken for the true *nutmeg liver,* occasioned by the moderate deposition of fatty matter: a microscopic examination will suffice at once to distinguish these morbid states from each other. The functions of the viscus are sometimes very little, if at all, disturbed by the fatty degeneration of its structure; the only inconvenience perhaps experienced by the patient being the distension of the belly, and a sense of fulness and weight on turning in bed from one side to the other, in consequence of the increased bulk of the liver.

The *fatty liver* occurs in different states of the body. 1. Corpulent inactive persons, who live full and grossly, and drink freely of heavy malt liquors.* Magendie found that dogs, which were fed almost exclusively

---

* Such persons, if they wish to reduce their unwieldiness, should abstain not only from greasy food and fermented liquors, but also from the use of much sugar, potatoes, and other substances whose chemical composition is nearly equivalent. "As sugar," remarks our author, "furnishes a material for respirawith oleaginous matters, as butter, lard, &c., died of inanition, although they became remarkably fat: in all, the liver was fatty. 2. In certain wasting diseases; but more especially—nay, almost peculiarly—in Phthisis. According to Louis, this change occurs in about one-third of phthisical patients: it is much more frequent in females than in males.* It is not very easy to account for its production. Some have supposed that, in consequence of the office of the lungs being gradually more and more interfered with, the elimination of the hydro-carbonaceous matters (of which fat is the most conspicuous) from the system is effected chiefly by some other organ, and that that organ is the Liver. But to this opinion it has been objected, that in Asthma and other diseases, in which the respiratory functions are very imperfectly performed, we never find the fatty liver; and, on the other hand, that this morbid change is occasionally met with, when the lungs are not at all affected. It has been found in persons who have died from Cancerous disease, from caries, from chronic dysentery,† and other wasting maladies.

The general inference, drawn by our author from these and other facts, is, that "in the process of wasting, the fat stored up in the body is largely taken up by the veins, so that it comes to be in excess in the blood, and is then laid hold of by the hepatic cells, which have a natural affinity for it. Fat is, without doubt, secreted in large quantity by the Liver, and by the sebaceous glands, whenever a large quantity of it finds its way into the blood." According to this view, the fatty degeneration of the liver is essentially not more a disease of this viscus, than Diabetes is a disease of the kidneys: these organs only serving to eliminate certain matters already formed within the system.

The "waxy liver" seems to be only a variety of the fatty liver, and need not detain us; and the sole reference that we shall make to the following section on "Scrofulous Enlargement and other kindred states," is to extract the following passage on the *treatment* of such maladies.

"When the enlargement of the liver is consequent on scrofula, our chief reliance must be on warm clothing; sea-air and bathing; a light nourishing diet, comprising a liberal allowance of animal food and wine; and the preparations of iodine and iron, separate or combined.

"When the health has been broken by the combined effects of syphilis and mercury, warm clothing, a tonic regimen, iodide of potassium, nitric acid, sarsaparilla, and guaiacum, are the appropriate remedies.

---

tion, which is soluble in the blood, it is acted on by oxygen much more readily than the insoluble fat, which is thus protected, and laid up in the system. Alcohol has a still stronger protecting power, for similar reasons."

Query—Does experience bear out this ingenious reasoning in reference to ardent spirits? Are those people, who are much given to their use, generally fat?

* There is often an accumulation of fat about the heart also, in Phthisis. This has been suspected to serve merely as a sort of compensation for the wasting of its muscular tissue; the heart requiring to have a certain amount of fulness and bulk, to admit of its cavities assuming the necessary changes of volume and position in the acts of systole and diastole.

† In persons, who have died of chronic Dysentery, we not unfrequently find that the *omentum* and *appendices epiploicæ* are unusually loaded with fat. Cruveilhier alludes to the frequent accumulation of fat about cancerous growths.

"In either case, the original malady is faulty assimilation, and, if we can remedy this, we shall probably, in most cases, if not in all, remedy the unnatural condition of the liver, and other secondary ailments.

"My own experience leads me to think highly of frictions with iodine ointment, long-continued, in such cases. I have several times seen an enlarged liver reduced to its natural volume by iodide of potassium and frictions with iodine, or, simply, by these frictions and saline purgatives. The matter deposited in the liver does not become organised, like the fibrine poured out in inflammation, and, if the general health mends, it may, in time, all pass off in the bile, or be removed by absorption." P. 254.

Dr. Graves has recorded, in his Clinical Medicine, several cases of enormously enlarged liver gradually subsiding under a course of treatment, persevered in, perhaps, from six months to two years.

The Section on *Gall-stones* is full of interesting matter. These substances are almost always formed at first by a portion of the bile becomirg so inspissated as to become solid; around this, as a nucleus—which is usually of a dark olive or black colour—is deposited Cholesterine, mixed with variable quantities of the colouring matters of the bile. They are usually formed in the gall-bladder; but occasionally in the hepatic ducts. The latter variety is generally much darker and more irregular on its surface—in consequence of the absence of any investing deposit of Cholesterine—than the ordinary kind. Some gall-stones—especially those which are solitary—are composed almost entirely of this substance (cholesterine), and are then nearly quite white, and of a crystalline appearance: they have a soapy feel, and burn in the flame of a candle with a bright flame. The number of gall-stones may vary from one to as many as two or three thousand.

"Gall-stones are very light, considering their size. When fresh from the gall-bladder, they usually sink, if placed in water. When they have been kept long, and are quite dry, most of them float, until they have imbibed a certain quantity of the water, when they sink slowly. Their specific gravity depends chiefly on the relative proportion of cholesterine and colouring matter. Cholesterine is lighter than water; the colouring matters of bile are heavier. The lightest gall-stones are therefore usually those which contain the largest proportion of cholesterine. The weight of gall-stones, especially when dry, will, of course, vary also with the character of their nuclei." P. 278.

Dr. Prout says that, when the passage of gall-stones is suspected, directions should be given to mix the fæces with water, on the surface of which the stones, if present, will be found floating. But this certainly will not always happen. "Dr. Watson has also recommended the adoption of this method of finding the stones, but he adds, 'I never but once succeeded in thus catching a concretion in the evacuations of a patient, whose symptoms had led me to search for it.'"

The formation of gall-stones appears to be much more frequent in women than in men; in consequence, probably, of their more inactive mode of life. Among men, it is chiefly among those whose pursuits are sedentary that they are met with: hence literary persons, prisoners, and bed-ridden invalids are most frequently the sufferers. Morgagni was of opinion that the causes of gall-stones are, in a great measure, the same as those of urinary calculi; and more recently, Dr. Prout has remarked that a tendency to the formation of gall-stones of cholesterine is frequently associated with a tendency to lithic acid deposits in the urine. In some cases, indeed, the symptoms of the urinary disturbance are so predominant as to disguise altogether for a time those of the hepatic suffering, so that the true nature of the attack has been mistaken, until perhaps the evacuation of the gall-stones ; after which the patient has speedily recovered.

The *symptoms of the passing of gall-stones* through the ducts into the duodenum are well described in the following passage:

"They generally come on suddenly, two or three hours after eating, with severe pain, like that of colic, in the region of the gall-bladder. The pain is not equable. There is a constant, dull, aching pain, which every now and then is interrupted by a paroxysm so excruciating that the patient bends himself double, or rolls about the floor, at the same time pressing his hands firmly against the pit of the stomach, which sometimes eases the pain. These severe paroxysms produced great exhaustion: the pulse becomes slow or weak, the face pallid, and the whole body covered with a cold sweat.

"Together with these symptoms, there is distressing nausea, and frequent vomiting. The matters vomited are very acid, and, as in all cases of repeated vomiting, while the common duct is not closed, are bitter.

"The severity of the symptoms, and the time they last, are of course very variable, depending on the number, and the form, and the size, of the stones that are passing, and on the previous state of the ducts. In some cases, the symptoms cease after an hour or two, or a still shorter time, and generally, *suddenly*, as the stone escapes into the duodenum,—and the complaint may be taken for mere hepatic colic. In other cases, where the stone is larger, or the passage is less free, or where many stones pass in succession, the symptoms may continue, with intervals of comparative ease, for several days." P. 288.

When the symptoms last long, the patient always becomes more or less jaundiced, and there is in many cases a marked tendency to the invasion of rigors, recurring at irregular intervals : the same thing is often observed in cases of Stricture of the Urethra, or when a renal Calculus descends along the ureter. Although the symptoms attending the passage of gall-stones are often most alarming, the case generally terminates favourably. Occasionally, however, it has proved fatal, either from the supervention of ileus, or from the rupture of the gall-bladder or ducts, and the consequent effusion of the bile into the abdominal cavity.

Chapter IV. is devoted to the consideration of *Cancer of the Liver*, and of *Hydatid Tumours*.

The first of these morbid growths is of much greater frequency than is commonly supposed. Our author says: "No serious organic disease of the Liver is, in this country—at least among people who have never drunk hard—so frequent as *Cancer*;" and Cruveilhier has observed—"of all the diseases of the liver, the most frequent and the most severe is, perhaps, the cancerous degeneration in the form of disseminated masses."

In the majority of cases, Cancer of the Liver is consequent upon the existence of cancer in some other part of the body, more especially in the stomach and mamma: in a few instances, indeed, the disease seems to be primarily developed in the hepatic parenchyma.

The greater liability of the liver than of any other organ (the lungs excepted) to be the seat of *disseminated cancer* as well as of *disseminated abscesses*, is chiefly attributable to the circumstance of the entire venous blood of the stomach and intestines having to pass through its substance, and to the consequent risk of various abnormal matters being detained in its capillary vessels. The *cells* of Pus, of Cancer, and of other morbid degenerations, are thus apt to become arrested in their course; and the result of such arrest must be to give rise to *foci* of purulent, cancerous, or other disease in different parts of the Hepatic parenchyma. Such being the case, we are at once prepared to expect that, whenever the stomach or any portion of the intestinal canal is affected with Cancer, the liver, ere long, will become the depository of some of the morbid and morbific germs. It is seldom that a *single* or isolated cancerous growth is found in this viscus. Usually there are scattered through its substance a great number—often hundreds—of round cancerous tumours; some of them so small as to be distinguished with difficulty, others of the size of a bean, or a walnut, or even of an orange.

"Cancerous tumors in the liver, as in other parts, differ much in firmness, vascularity, and colour, in different cases. Sometimes, the tumors are white and fibrous, or, as it is termed, *scirrhous;* but far more frequently, especially when numerous, they are soft, or *medullary*. Instances are now and then met with, in which, in the same liver, some tumors are hard, and others soft.

"Soft cancer presents the same varieties in the liver, as in other organs. Most commonly the cancerous mass contains but few vessels, and is pulpy and whitish, or of a greyish-white—presenting that striking resemblance to brain rather softened, which led Laennec to apply to it the term, *encephaloid.* In other cases, the tumors are extremely vascular, presenting the appearance known as *fungus hematodes.* In others, again, they are *melanotic.* Indeed, every variety of cancer, except *gelatiniform,* or *colloid* cancer, has been met with in this organ.

"The colour of melanotic tumors of the liver varies, according to the quantity of pigment granules which they contain. In the same liver tumors may sometimes be found of every shade from light brown to black." P. 302.

A liver, which is the seat of numerous masses of Cancer, usually becomes very much enlarged, so that it may reach down considerably below the false ribs, and even down to the brim of the pelvis; and, as its surface is sometimes tuberculated with the cancerous growths, it may then be felt irregular and knobby on examination of the abdomen. In a few rare instances of the disease, the bulk of the viscus is diminished. In many cases of cancer of the liver, there is more or less of Ascitic effusion present; but the quantity of the effusion is very seldom so great as in *cirrhosis* of this organ. Not unfrequently, the patient is jaundiced at the same time.

"The most significant symptom" (of cancer), says our author, "is enlargement of the liver. When this comes on in the middle period of life, and especially when it is progressive, and when other conditions that may equally give rise to it, are wanting,—when there is no obstacle to the circulation in the chest, when the patient is not consumptive, and when his habits have not been such as to lead us to suspect that he may have cirrhosis,—it affords, of itself, strong presumption of the presence of cancerous tumors. When the liver is of very great size, and its surface can be felt to be nodulous or uneven, there is no longer room for doubt.

"Another symptom which is of very frequent occurrence, and which may help us to distinguish this disease from some others in which the liver is likewise enlarged, is constant pain and tenderness." 326.

Whenever there is good reason to suspect the cancerous nature of an enlargement of the Liver, we should very carefully avoid having recourse to large or repeated doses of *mercury :* they can do no possible good ; and may rapidly exhaust the patient's strength, and aggravate, most probably, at the same time, the local malady.

*Hydatid tumours* are very frequently found in the Liver; indeed more so in this than in any other organ of the body. Their contents are usually transparent and limpid as water ;* but, in some cases, the sac or cyst is filled with a thick matter, having the appearance of glazier's putty or plaster, mixed with fragments of dead hydatids. This matter is found to consist of phosphate of lime, with perhaps the addition of a little carbonate, and an albuminous substance. When there is an Hydatid tumour in the liver, there are frequently similar tumours at the same time either in the Lungs, in the Spleen, or in some part of the Mesentery. In some cases, thousands of hydatids have been found in the abdomen, under the peritoneum, and between the folds of the mesentery. It is often difficult to determine in what part or organ the primary and parent tumour was formed. Dr. Budd seems to regard the Liver as generally the "point de depart;" for he says :—

" The constancy with which hydatid tumors in the liver are associated in one case with hydatid tumors in the lungs only; in another, with hydatid tumors in the spleen, or in the mesentery only, strongly favours the supposition, that a tumor of the liver may, by the escape of germs into a branch of the hepatic or of the portal vein, or into one of the lymphatics, lead to secondary tumors in the lungs, or in the liver itself, or between the folds of mesentery. In such cases, too, there is generally one tumor in the liver, which, from its greater size, from the greater thickness of its coats, and from other marks of age, looks like the parent of the rest. In a large proportion of such cases, this patriarchal-looking tumor presents ulceration, or other marks of disease, on the inner surface of the sac." 354.

There is the objection however to this hypothesis—as our author himself remarks—that we must suppose that the hydatidic germ or cell passes backwards, against the current of the blood, in the veins that go to form the *vena portæ*. The chief argument in its favour is the circumstance, that what is apparently the parent cell (judging from its size, and the thickness of its parietes), is generally found in the Liver.

Hydatid tumours, similar to those we find in the human subject, are very common in sheep, and in other herbivorous animals : they have never been discovered in animals of any other class. The Liver is the organ that is most frequently their seat. It has been said, that if one sheep in a flock is affected with them, all the others will become so, more or less. They are especially frequent in wet seasons, and when the sheep are fed in ill-

* This fluid is however most irritating to serous membranes, which it may happen to touch; as sometimes takes place by the bursting of a hydatidic cyst, and the effusion of its contents into the peritoneal cavity. The inflammation thus induced may be as rapid and intense, as that occasioned by the bursting of the gall-bladder. Mr. Cæsar Hawkins has related several cases, in which the fluid of hydatid cysts in the mamma and other parts was very irritating, and caused sloughing and fungoid ulceration.

drained pastures. There are many points in the history of Hydatids of the Liver that we could have wished to have alluded to; but our limits forbid us, and we must therefore refer the curious reader to Dr. Budd's most interesting narrative for instruction. The single remark that we can find room for is, that the only medicines, which have been supposed to arrest the growth of these Entozoa, are the *iodide of potassium* and *common salt*.* It is well known that Hydatidic tumours have been, in some instances, successfully punctured and emptied of their contents. In other cases, however, very troublesome consequences have ensued upon this simple operation. Mr. Cæsar Hawkins dissuades the surgeon from interfering with them, unless the urgency or gravity of the existing symptoms seems to call for immediate relief.

We must now draw our notice of Dr. Budd's excellent work to a close. Its general character, as the reader will have perceived, is rather *pathological* than *therapeutic;* but as every improvement in our knowledge of the organic changes, which accompany the diseases of any part, must eventually tend to introduce a more rational and successful method of treating them, we cannot too strongly recommend the diligent study of this volume to all physicians. It is the first comprehensive attempt, that we know of, to apply the recent discoveries in the minute structural anatomy of the liver to the elucidation of its morbid states, and cannot fail to rank the name of its author among the most enlightened pathologists and soundest practitioners of the day.

* It is worthy of notice that Hydatid tumours are extremely rare among sailors. Mr. Bush, who has been surgeon to the hospital on board the Dreadnought almost from its first establishment, has met with only one case among the many thousand patients who have been admitted since that time. It appears also that by far the most efficacious remedy, next to a removal to a dry pasturage, for the *rot* in sheep—a disease depending upon the liver and gall-bladder of the animal becoming infested with those parasites known by the name of *liver-flukes*, Distoma hepaticum and D. lanceolatum—is the free use of common salt with the food. The curious reader is referred to the Treatise on the Sheep, in the Library of Useful Knowledge, for the best account of this disease.

